# Language and turn-taking in schizophrenia spectrum disorders

**DOI:** 10.1192/j.eurpsy.2022.1971

**Published:** 2022-09-01

**Authors:** L. Dusi, V. Lucarini, F. Cangemi, J. Lucchese, F. Giustozzi, F. Magnani, C. Marchesi, K. Vogeley, M. Grice, M. Tonna

**Affiliations:** 1 University of Parma, Psychiatry Unit, Department Of Medicine And Surgery, Medical Faculty, Parma, Italy; 2 Université de Paris, Équipe Physiopathologie Des Maladies Psychiatriques, Umr 1266 Ipnp Inserm, Paris, France; 3 University of Cologne, Ifl‑phonetics, Cologne, Germany; 4 Azienda Unità Sanitaria Locale di Parma, Department Of Mental Health, Parma, Italy; 5 University of Cologne, Department Of Psychiatry And Psychotherapy, Medical Faculty, Cologne, Germany; 6 Research Center Jülich, Cognitive Neuroscience (inm‑3), Institute Of Neuroscience And Medicine, Jülich, Germany

**Keywords:** schizophrénia, language, Conversation, turn-taking

## Abstract

**Introduction:**

Language and conversation are deeply interrelated: language is acquired, structured, practiced in social interactions and linguistic resources (specifically syntactic, prosodic and pragmatic aspects) contribute to finely tuning turn-taking. Nevertheless, most studies focused on verbal aspects of speech in schizophrenia, with scant attention to their relation to conversation, where language is experienced at most.

**Objectives:**

The present study was aimed at investigating a possible association between language impairment and conversational characteristics in a sample of clinically stable patients diagnosed with schizophrenia (N = 35, ages 18-65).

**Methods:**

A spontaneous speech sample was recorded. For the assessment of language skills, the Scale for the Assessment of Thought, Language and Communication (TLC) and the Clinical Language Disorder Rating Scale (CLANG) were used, while conversational variables were extracted with an innovative method of semi-automatic analysis. The possible associations were investigated through the Pearson Correlation.

**Results:**

Figure 1 represents graphically the correlational matrix between conversational variables and linguistic scale scores. In the heatmap, blue means negative and red positive correlations, the stronger the colour, the larger the correlation magnitude. Moreover, the significant associations are indicated with stars.

**Figure:**
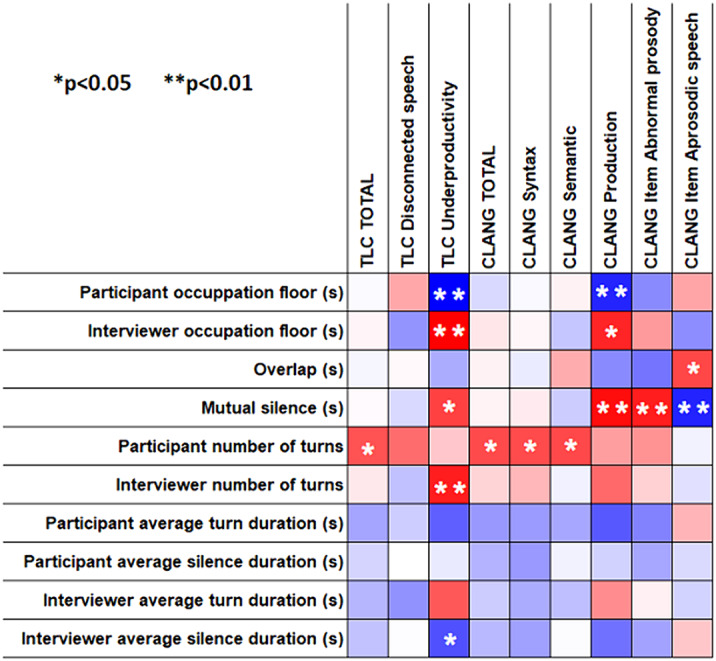

**Conclusions:**

The results suggest that in schizophrenia spectrum disorders the disturbances of language, at a syntactic, prosodic and pragmatic level, have significant impact on communicative interaction.

Thus, conversation analysis might be a promising method to quantify objectively communicative impairment with the benefit of representing an ecological assessment, examining the performance of patients in the real situation of language use, which is social interaction.

**Disclosure:**

No significant relationships.

